# Supplementary material for: Community Co-Design of Regional Actions for Children’s Nutritional Health Combining Indigenous Knowledge and Systems Thinking
Source: Int J Environ Res Public Health. 2022 Apr 19;19(9):4936. doi: 10.3390/ijerph19094936 (PMC9106006; doi:10.3390/ijerph19094936)

Supplementary materials

Supplementary figure 1. Example of a causal loop diagram drawn in the community workshops (Adult stakeholder table)

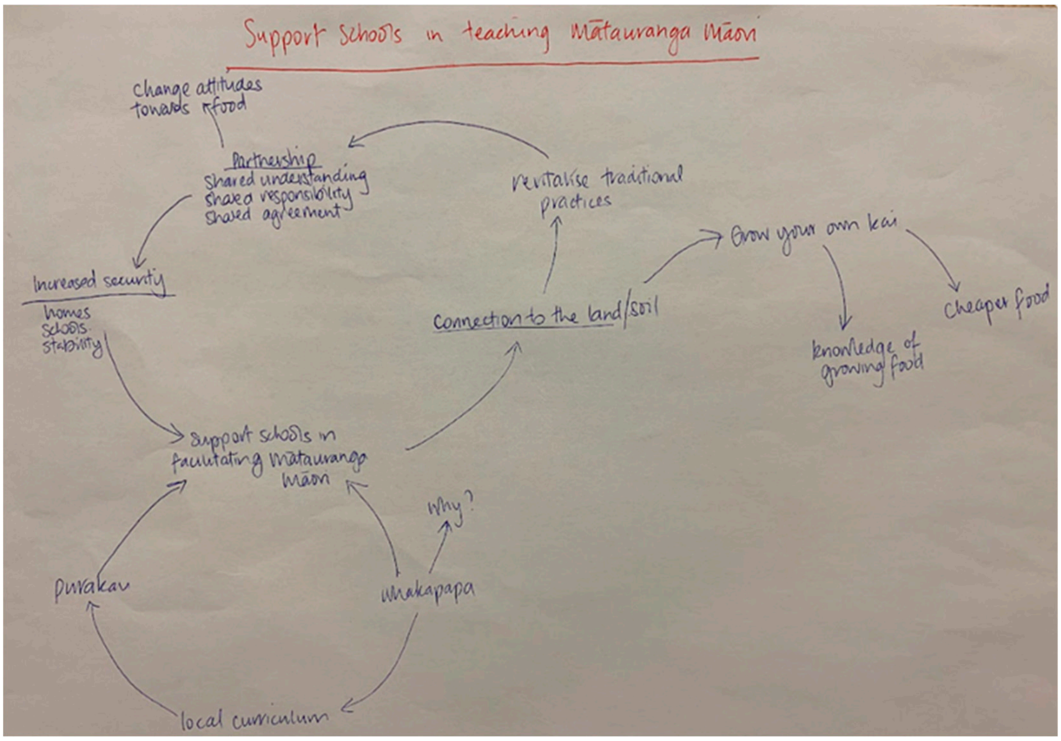

Supplement: Supplementary file 1 [file ijerph-19-04936-s001.zip › Supplementary materials.pdf]
